# Expression patterns of conjunctival mucin 5AC and aquaporin 5 in response to acute dry eye stress

**DOI:** 10.1371/journal.pone.0187188

**Published:** 2017-11-07

**Authors:** Dhruva Bhattacharya, Li Yu, Mingwu Wang

**Affiliations:** 1 Department of Ophthalmology and Vision Science, University of Arizona College of Medicine, Tucson, Arizona, United States of America; 2 Shenzhen Key Laboratory of Ophthalmology, Jinan University Shenzhen Eye Hospital, Shenzhen, Guangdong, PR China; 3 Shenzhen Ocular Trauma and Stem Cell Differentiation Service, Shenzhen, Guangdong, PR China; 4 Shenzhen University College of Optometry, Shenzhen, Guangdong, PR China; 5 NeuVision Medical Institute, Tucson, Arizona, United States of America; University of California, Merced, UNITED STATES

## Abstract

The relationship between aquaporin (AQP) 5 and mucin (MUC) 5AC in the conjunctiva was investigated in response to acute dry eye (DE) stress. A mixed-mechanism rabbit DE model, in which the main lacrimal gland, Harderian gland, and nictitating membrane were resected, was further explored in this study. Conjunctival impression cytology specimens were harvested before excision (BE) and up to 3 months after excision (AE) in 8 (16 eyes) male New Zealand White rabbits, and immunoblotting was employed to assess the expression of AQP5 and MUC5AC. It was observed that AQP5 and MUC5AC showed a positive, synchronous expression pattern with progressive upregulation at protein level up to 2 months AE. At 3 months, the expression of both proteins decreased, but was still higher than that of BE. Such a synchronous relationship was further observed in mouse conjunctiva epithelium primary cells under hyperosmotic condition. Moreover, the co-immunoprecipitation of AQP5 and MUC5AC suggested a possible physical interaction between the two molecules. Our data indicates that conjunctival AQP5 and MUC5AC act synchronously in response to acute DE stress.

## Introduction

The tear film (TF), composed of water, lipids, mucin and antimicrobial substances, provides a barrier to protect the cornea from exogenous microbial/chemical agents and environmental insults, thus facilitating the maintenance of a smooth refractive surface necessary for clear vision [1]. The TF mucin lubricates/protects the superficial ocular epithelium (retards fluid evaporation) and anchors the TF to ocular surface [2]. The secreted gel-forming mucin defines the rheological properties of the mucus layer on epithelia. MUC5AC is a major gel-forming mucin secreted by conjunctival goblet cells embedded within mucosal epithelium or submucosal glands [1]. MUC5AC acts as a surfactant for the ocular surface, allowing an evenly spread TF to wet the hydrophobic epithelium [3]. Blinking of eyelids distribute conjunctival mucin over the ocular surface to remove debris/pathogens [4]. The glycosylation/secretion of ocular surface mucin along with a correlated goblet cell density may be altered in disorders such as dry eye (DE) [5–9]. In fact, severe DE shows a total loss of conjunctival goblet cells with end-stage keratinization [10]. DE patients with Sjögren's syndrome (SS) have been shown to have decreased goblet cell-derived MUC5AC content in their tears [9].

Aquaporin (AQP) water channels have been implicated in osmotically driven water transport across cell plasma membrane to maintain tear volume and osmolarity in ocular surface [11,12]. The AQPs 3, 4 and 5 have been detected in conjunctival epithelium but only AQP4 and AQP5 function as water selective transporters [13–15]. We previously demonstrated the potential role of AQP5 in the maintenance of ocular surface fluid balance in rabbits [13]. Significant upregulation of AQP5 was observed in the conjunctival epithelium 1 to 3 months after bilateral surgical excision of the main lacrimal gland (LG), Harderian glands (HG), and nictitating membrane (NM) [13].

The expression of AQP5 and MUC5AC has reportedly been either positively or negatively correlated in the respiratory track epithelium. Chen et al. reported that silencing AQP5 expression enhanced MUC5AC synthesis in human lung adenocarcinoma cell line [16]. But later in the same cell line, they showed that AQP5 overexpression increased MUC5AC expression [17,18]. Whereas decreased AQP5 expression and overproduction of MUC5AC have been reported in airways of patients with chronic obstructive pulmonary disease (COPD) [19].

Interestingly, diquafosol sodium and rebamipide promote secretion of both tear and mucin in DE patients [20,21]. The P2Y2 receptor is distributed within the ocular surface, including goblet cells, palpebral and bulbar conjunctival epithelium. Adenosine triphosphate (ATP) or uridine triphosphate (UTP) activates conjunctival P2Y2 receptor to promote secretion of water and mucin. Diquafosol has P2Y2 receptor agonist activity equivalent to that of UTP. Rebamipide is a quinolinone derivative with a mucin secretagogue activity [20,21]. Similarly, JBP485 (placental extract-derived dipeptide) promotes both increased MUC5AC expression (through goblet cells in conjunctival epithelium) and aqueous tear secretion (through ocular surface epithelium) in rabbits [22]. JBP485 accelerates tear secretion in a rabbit model in a dose-dependent manner [22]. Taken together, these reports imply that close regulatory mechanism exists between AQP5 and MUC5AC at the ocular surface, similar to that in the airway.

In the present study, we further explored the expression pattern of AQP5 and MUC5AC in the conjunctival epithelium of rabbits before excision (BE) and after excision (AE) of the main LG, HG, and NM as well as in mouse conjunctival primary cells.

## Materials and methods

### Experimental animals and ethics statement

This study was done using male New Zealand white rabbits (N = 8, 16 eyes, Harlan Sprague Dawley, Indianapolis, IN, USA) weighing 2.0–2.5 kg. The rabbits were reared under standard laboratory conditions (22 ± 2**°**C, 40% ± 5% relative humidity, and a 12-hour light-dark cycle) with free access to food and water throughout the experiment [13]. The study was conducted in compliance with the Tenets of the Declaration of Helsinki and ARVO statement for the use of animals in ophthalmic and visual research. The protocol was approved by the University of Arizona (Tucson, AZ, USA) Institutional Animal Care and Use Committee (protocol# 14–511). All surgeries were performed by Dr. Mingwu Wang.

The anesthesia was inducted in the rabbits for the eye surgery with an intramuscular injection of Ketamine (25 mg/ kg; Henry Schein, New York, NY, USA) and Xylazine (5 mg/kg; Akorn, Inc., Decatur, IL, USA) and each rabbit was intubated with an uncuffed endotracheal tube. The general anesthesia was maintained by 1% to 4% Isoflurane gas with 1.5 L/min oxygen flow during the entire surgical procedure of the rabbits. Buprenorphine sustained release (0.3 mg/kg; Hospira, Lake Forest, IL, USA) was given subcutaneously for perioperative pain management. Tobradex ophthalmic ointment (Alcon, Puurs, Belgium) was administered topically at the surgical wounds three times a day for 7 days, and Enrofloxacin (10 mg/kg; Bayer, Pittsburgh, PA, USA) was given subcutaneously once daily for 3 days as prophylactic anti-infection. At the end of the study the rabbits were anesthetized (with Ketamine and Xylazine) as per the protocol mentioned above and then sacrificed with an intra cardiac injection of Beuthanasia-D Special (C-3N) (Merck Animal Health, NJ, USA).

The black Swiss mice, 4 to 22 weeks old (Taconic Farm, USA) were anesthetized with an intramuscular injection of Ketamine and Xylazine as per the protocol mentioned above. Next, the mice were sacrificed by cervical dislocation and the conjunctiva tissue was removed for further experiments.

### Operative procedure and evaluations of rabbits

The detailed surgical protocol for resection of main LG, HG, and NM from rabbit eyes has been previously published [13,23]. Post surgery, the rabbit eyes were evaluated sequentially by corneal fluorescein test, rose Bengal staining, conjunctival impression cytology (CIC), and Schirmer tests as previously discussed [13,23]. The rabbits were evaluated BE as a baseline and AE monthly for up to four months [13].

### Conjunctival impression cytology of rabbits

CIC was performed as per the published protocol [13]. The filter paper discs were peeled off and immediately placed in either, Trizol solution (Invitrogen, CA) for RNA isolation or 100 μl of radio immunoprecipitation assay (RIPA) buffer (Teknova, CA, USA) for protein isolation.

### Isolation and culture of mouse conjunctival primary epithelial cells

The mouse conjunctival primary epithelial cells were isolated and cultured using a modified protocol as published previously [24,25]. The conjunctiva tissue, excised under a dissection microscope from both eyes of each mouse, was placed in Hank’s balanced salt solution (Lonza, Walkersville, MD, USA) containing 3× (300 mg/ml) penicillin-streptomycin (Invitrogen, CA, USA). The conjunctiva fragments were finely minced into smaller pieces (~65 to 90 tissue explants obtained from each animal) and placed in Petri dishes containing cell culture media [RPMI-1640 medium (Lonza, Walkersville, MD, USA) supplemented with 10% heat-inactivated fetal calf serum (Thermo Fisher Scientific, Waltham, MA, USA), 1mM sodium pyruvate, 10mM HEPES, 100*μ*g/mL penicillin-streptomycin, and 1× nonessential amino-acid mixture (Lonza, Walkersville, MD, USA)]. Explants were grown under 5% CO_2_ in a humid incubator at 37**°**C and fed with the culture media every 48 hr. The cells were grown from the tissue explants for 14 days until 85% confluence was achieved. The explants were then removed by trypsin (Thermo Fischer Scientific, MA, USA) treatment and the conjunctival primary epithelial cells were re-plated for further experiments.

### Hyperosmolar treatment of mouse conjunctival epithelial primary cells

The mouse conjunctival epithelial primary cell cultures of ~70% confluence (5×10^5^ cells/well) were washed thrice with 1× PBS and further cultured in a serum-free medium (RPMI media without FBS) for 24 hours before any experiment. The epithelial cells were then cultured for an additional 24 hours in a serum free RPMI media of different osmolarities (350 and 400 mOsm) by adding appropriate amount of sodium chloride [26]. The media osmolarity was verified by an advanced^®^ Micro-Osmometer Model 3300 (Advanced Instruments, Inc. Massachusetts, USA) prior to the experiments. In addition, some cells were grown separately in isosmotic medium as time matched control. Finally, the cells were washed, re-suspended in 1× PBS, and centrifuged at 14000 RPM for 10 min at 4**°**C. The cell pellets collected were processed for RNA and protein isolation.

### RNA isolation and cDNA synthesis

Total RNA was isolated from the cells in Trizol solution according to manufacturer’s instructions (Invitrogen, CA, USA). RNA concentrations were estimated as per published protocol [13]. The first strand of cDNA was synthesized in a final volume of 20 μl reaction mix. First, 1 μl of Oligo (dT) 20 Primer and 10 mM dNTPs was added to 500 ng total RNA in final volume of 13 μl. The mix was heated to 65°C for 5 min and incubated on ice for 2 min. Then, 5× First-Strand Buffer (4 μl), 0.1 M DTT (1 μl), RNaseOUT^™^ Recombinant RNase Inhibitor (1 μl) and SuperScript^®^ III Reverse Transcriptase (1 μl) were added. The reaction mix was incubated at 50°C for 60 min and the reaction inactivated by heating at 70°C for 15 min. Further, to remove RNA complementary to the cDNA, 2 units of RNase H was added and incubated at 37**°**C for 20 min. The cDNA synthesis reagents were purchased from Invitrogen, CA, USA.

### Reverse Transcriptase-Quantitative polymerase chain reaction (RT-qPCR)

The RT-qPCR reactions were set using SYBR^®^ Green PCR Master Mix (Applied Biosystems, Foster City, CA, USA) according to manufacturer’s instructions. The primer sequences for the genes studied using qPCR are listed in Table 1. The RT-qPCR was performed on StepOnePlus^™^ Real-Time PCR System (Applied Biosystems) with the following cycling conditions: 10 min 95°C; 40 cycles of 15 sec 95°C, and 30 sec 60°C. A melting curve analysis was performed at the end of each PCR by gradually increasing the temperature from 60 to 95°C while recording the fluorescence. A single peak at the melting temperature of the PCR-product confirmed primer specificity. To compare between different runs, a fixed fluorescence threshold for derivation of cycle threshold (Ct) value for all runs was used. Three technical replicates were performed for each biological specimen to evaluate the relative quantification.

**Table 1:** Primer sets used for quantitating gene expressions by RT-qPCR.

Gene	Forward primer	Reverse primer	NCBI Accession number/ Ensembl Gene number
IL-1β	GCTGCTTCCAAACCTTTGAC	AGCTTCTCCACAGCCACAAT	NM_008361.3
TNF-α	TGTGCTCAGAGCTTTCAACAA	CTTGATGGTGGTGCATGAGA	Benevides et al. (2013) [27]
Gapdh	GAAGGGCTCATGACCACAGT	GGATGCAGGGATGATGTTCT	GU214026.1
AQP5	GCCATCTTGTGGGGATCTAC	CCCAGAAGACCCAGTGAGAG	NM_009701
Muc5ac	CACTGGAGCTGGATGTCAGA	ACAACACAGCCTCCATTTCC	NM_010844.1
K4	CAGGCTCTCATGAACGTCAA	TACCGAGATGCTCACAGCAC	ENSMUSG00000059668
K12	TGACGAGAGCTCATCCCTTT	ATGAGACCACTTCGCCATTC	ENSMUSG00000020912

### Relative quantification of mRNA level

Relative quantification (mean fold change) of gene expression were calculated in the mouse conjunctival primary epithelial cells samples after hyperosmotic treatment (HT) and no HT using 2^-ΔΔCt^ method, where ΔΔCt = (Ct_Gene_- Ct_Gapdh_) _HT_—(Ct_Gene_- Ct_Gapdh_) _no HT._ The fold change in gene expression was relative to the internal housekeeping gene, Gapdh. Difference between Ct for genes and Gapdh mRNA in each sample was used to calculate level of target mRNA relative to that of Gapdh mRNA in the same sample [13].

### Immunoblotting

**Fig 1:**
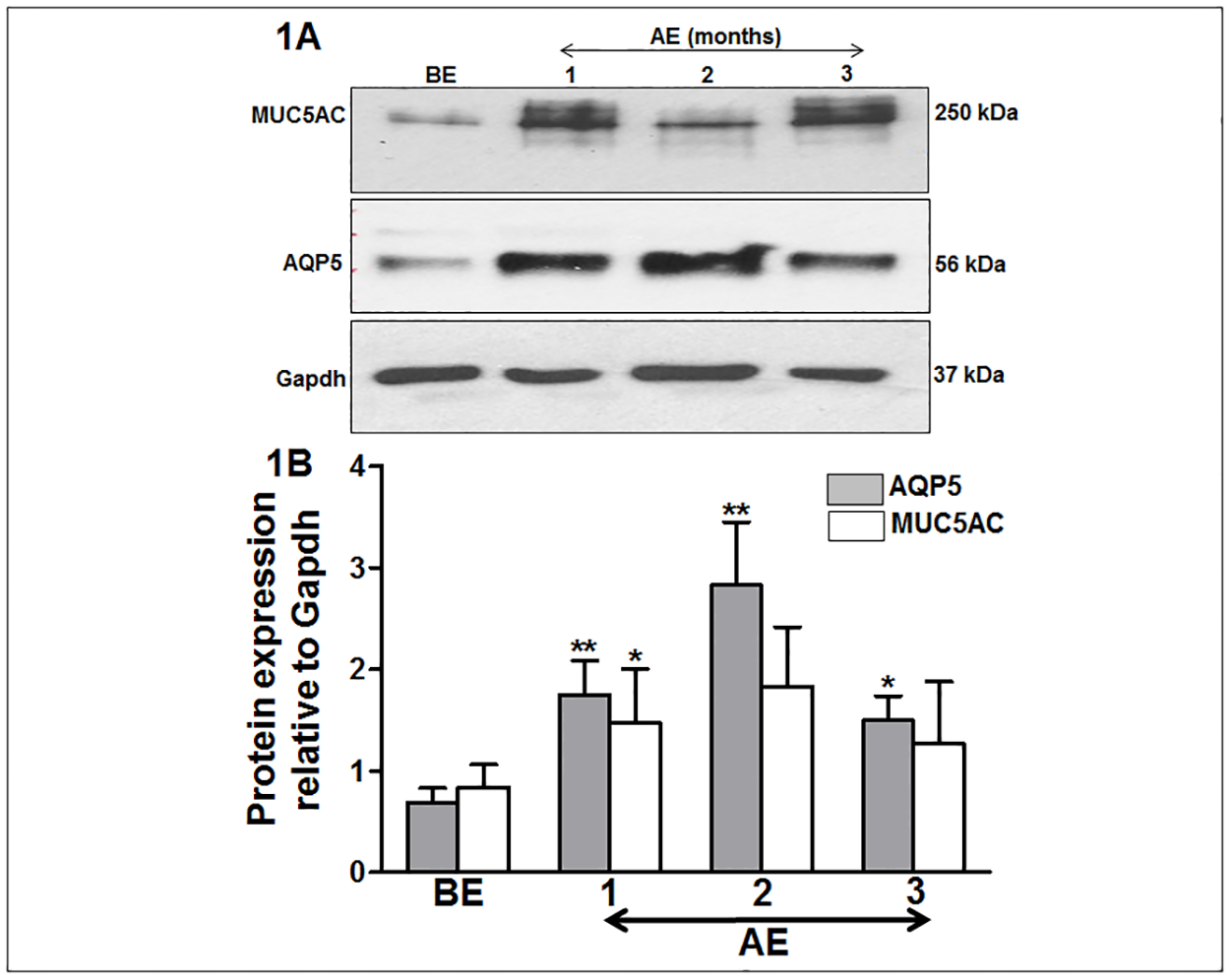
Increase in conjunctival AQP5 and MUC5AC expression in rabbits after surgery. In comparison to before excision (BE), the AQP5 protein increased significantly at 1, and 2 months after excision (AE), and then decreased slightly at the end of 3 months AE. The MUC5AC protein expression followed similar pattern of upregulation, demonstrating a synchronous relationship with AQP5. The protein (immunoblot) signals of AQP5 and MUC5AC were presented relative to the internal control “Gapdh” signal.

Total cell lysate proteins were isolated from the rabbit CIC samples in RIPA buffer with 1× HALT protease and phosphatase inhibitor single use inhibitor cocktail (Thermoscientific, Rockford, IL, USA) by incubating on ice for 30 min. Protein concentration was determined by Pierce^™^ BCA Protein Assay Kit (Thermo Fisher Scientific, NY, USA) as per manufacturer’s instruction. The samples were mixed with Laemmli sample buffer (Bio-Rad laboratories, Inc. Hercules, CA, USA) containing β-mercaptoethanol, heated at 42°C for 30 min, immuno-blotted, and analyzed as per published protocol [13]. The rabbit monoclonal primary antibodies to AQP5, MUC5AC (Abcam, Cambridge, MA, USA) and Gapdh (Santa Cruz Biotechnology, CA, USA) were used at appropriate dilutions. The protein expression of AQP5 and MUC5AC were analyzed using “Image J” software [28]. The protein (immunoblot) signals of AQP5 and MUC5AC were presented relative to the internal control “Gapdh” signal (Fig 1A).

### Co-immunoprecipitation

Freshly frozen normal rabbit conjunctival tissue was homogenized in RIPA buffer containing protease/phosphatase inhibitor to prepare the conjunctival tissue protein lysate. Next, 50 μl (1.5 mg) re-suspended Dynabeads^®^ protein A (Life technologies, CA, USA) was transferred to a 1.5 ml tube, placed on the magnet (separate beads from solution), and the supernatant removed by pipetting. The tube was then removed from the magnet, 5 μg of primary monoclonal antibody (Ab) to AQP5 (diluted in 200 μl 1×PBS with Tween^®^-20) was added, incubated with rotation for 15 min at room temperature, and placed on the magnet again to remove the supernatant. Further, the tube was removed from the magnet and the beads-Ab complex was re-suspended in 200 μl 1×PBS with Tween^®^-20. Next, the tube containing beads-Ab complex was placed on the magnet to remove the supernatant. The rabbit conjunctival tissue lysate (500 μg) was mixed with the beads-Ab complex, incubated with rotation for overnight at 4°C to allow antigen(s) to bind to the beads-Ab complex. Later, the tube was placed on the magnet and the supernatant was removed. The beads-Ab-Ag complex was then washed thrice with 200 μl of 1 × PBS Buffer (with 100 mM NaCl and Tween^®^-20) for each wash, separated on the magnet between each wash, removed supernatant, and re-suspended by pipetting. The beads-Ab-Ag complex was finally re-suspended in 50 μl Lamaelli sample buffer, heated for 5 min at 95°C, resolved on denaturing SDS-PAGE as previously published and immunoblotted for MUC5AC using the primary rabbit monoclonal antibodies MUC5AC (Abcam, Cambridge, MA, USA) at a dilution of 1:200. Similarly, the immunoprecipitation of MUC5AC was done using primary monoclonal Ab to MUC5AC (diluted in 200 μl 1×PBS with Tween^®^-20) as per above protocol [13]. The AQP5 immunoblot was using primary rabbit monoclonal antibodies AQP5 (Abcam, Cambridge, MA, USA) at a dilution of 1:200.

### Data analysis and statistics

Data in figures are presented as mean Standard Error Method, the bars representing standard errors. Statistical significance between 2 groups (BE and AE) was evaluated using unpaired two-tailed T test. A probability of P = 0.05 was considered significant (where applicable, *P < 0.05, **P < 0.01, ***P < 0.001).

## Results

### Expression of AQP5 and MUC5AC in rabbit conjunctival epithelium

The immunoblotting against AQP5 in the total protein isolated from the rabbit CIC showed the presence of the dimeric form of AQP5 (size ~ 54 kDa) (Fig 1A). Detection of the dimeric form of integral membrane protein is a known characteristics [15,29,30]. The AQP5 protein level increased at 1 month AE and doubled at 2 months when compared to BE (Fig 1B). Though at 3 months AE the AQP5 level slightly reduced, yet it was still elevated compared to that of BE (Fig 1B). Immunoblot study against MUC5AC detected the expected protein size of 250 kDa (Fig 1A). The MUC5AC protein level showed a similar expression pattern as AQP5. The MUC5AC level increased at 1 month AE and doubled at 2 months compared to BE (Fig 1B). At 3 months AE the MUC5AC level decreased as well (Fig 1B).

### Interaction between conjunctival epithelium AQP5 and MUC5AC

**Fig 2:**
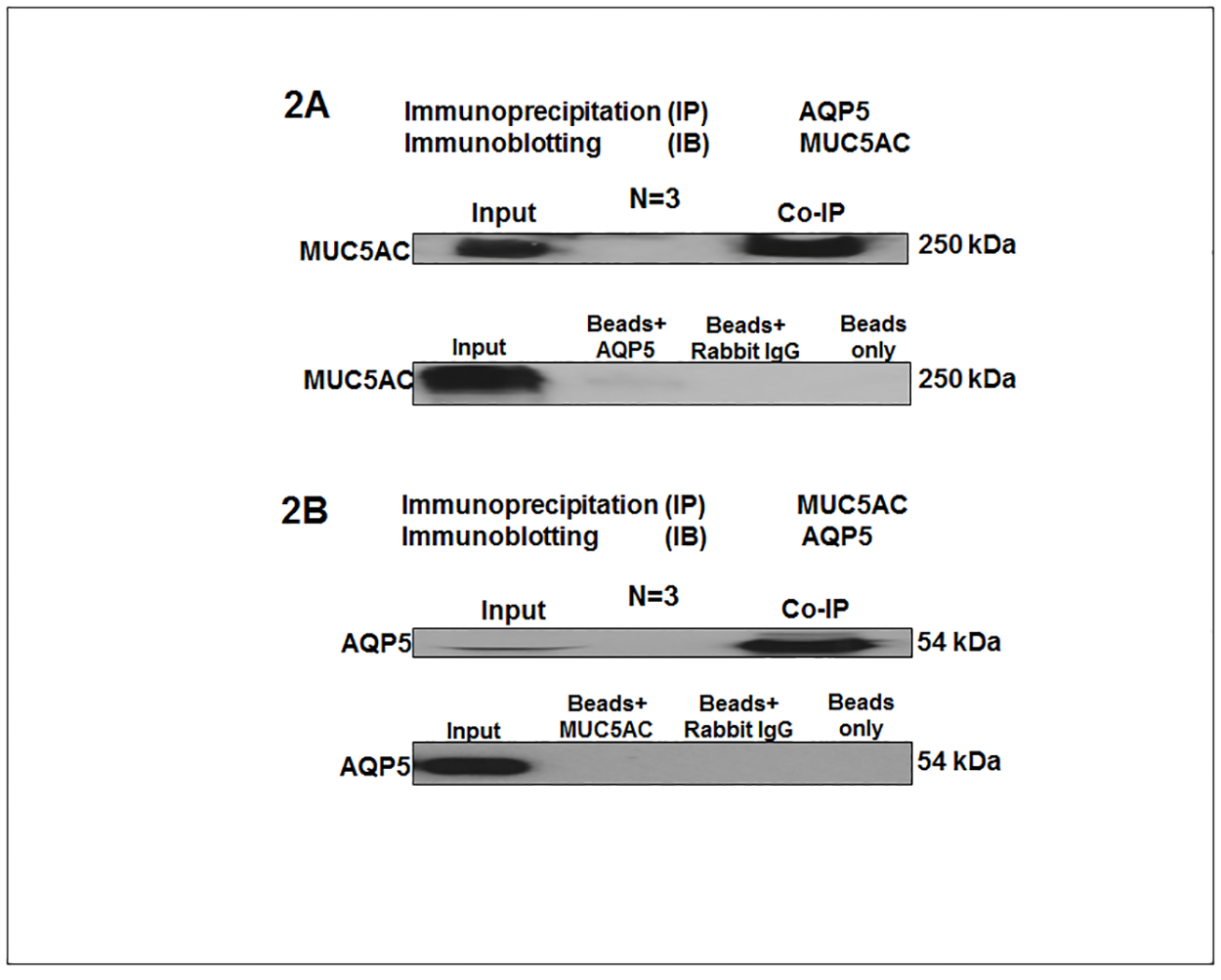
Co-immunoprecipitation (CoIP) of AQP5 and MUC5AC. (2A) The detection of MUC5AC in Ab-Ag complex by immunoprecipitation of AQP5 from rabbit conjunctival tissue protein lysate with AQP5 antibody bound to Dynabead^®^ Magnetic beads coupled Protein A. Input: The normal rabbit conjunctival tissue protein lysate (Positive control for MUC5AC); Co-IP: MUC5AC protein signal detected in the Co-IP Ab-Ag complex by AQP5 antibody; Negative controls: Beads+AQP5 (magnetic beads incubated with only the AQP5 antibody); Beads + rabbit IgG (magnetic beads incubated with only the IgG antibody); Beads only (magnetic beads alone incubated in the reaction buffer). (2B) The CoIP of AQP5 by immunoprecipitation of MUC5AC from rabbit conjunctival tissue protein lysate with MUC5AC antibody bound to Dynabead^®^ Magnetic beads coupled Protein A. Input: The normal rabbit conjunctival tissue protein lysate (Positive control for AQP5); Co-IP: AQP5 protein signal detected in the Co-IP Ab-Ag complex by MUC5AC antibody; Negative controls: Beads+ MUC5AC: Beads + rabbit IgG; Beads only.

Immunoprecipitation with anti-AQP5 antibody pulled down MUC5AC (Fig 2A) from the rabbit conjunctival protein lysate. Vice versa, anti-MUC5AC antibody pulled down AQP5 (Fig 2B) from the rabbit conjunctival protein lysate. This suggests a potential interaction between AQP5 and MUC5AC within one protein complex.

### Mouse conjunctival epithelial primary cells as an in vitro dry eye model

**Fig 3:**
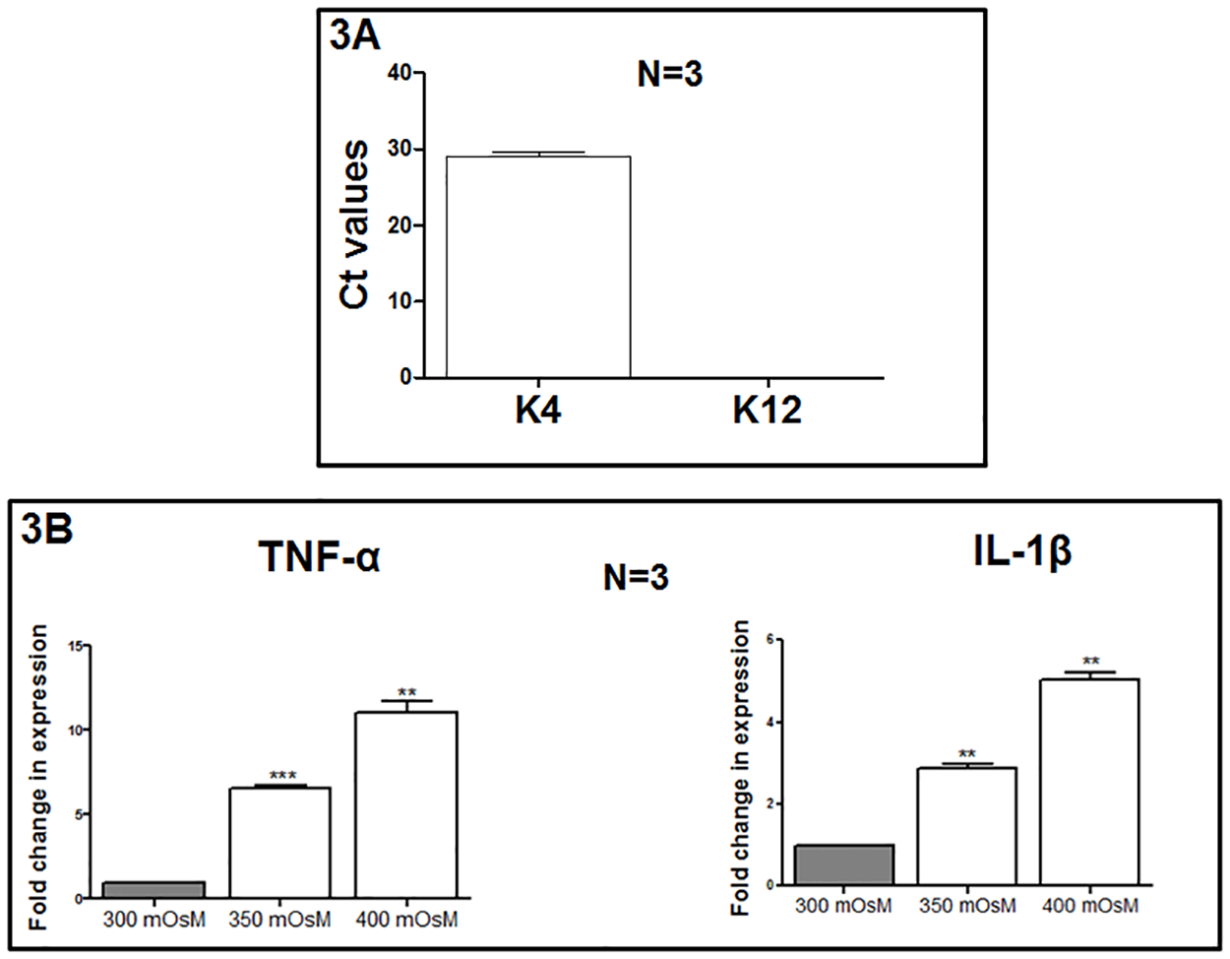
Characterization and hyperosmotic treatment of the mouse conjunctival epithelium primary cells. The confirmation of the origin of primary epithelial cells was done by assessing the levels of cytokeratin 4(K4) and cytokeratin 12(K12) by RT-qPCR (Fig 3A). Under hyperosmotic conditions (350 mOsm and 400 mOsm) for 24h, significant increase in the mRNA expression of proinflammatory cytokine markers, TNF-α and IL-1β, was noted (Fig 3B).

The mouse conjunctival epithelial primary cells were characterized by cytokeratin (K) 4 (conjunctiva specific) and cytokeratin (K) 12 (cornea specific) markers [31,32]. The K4 markers showed a significant Ct value where as K12 marker could not be detected in these cells. This confirms that the primary epithelial cells were derived from mouse conjunctiva (Fig 3A). Further, hyperosmolar (350 mOsm and 400 mOsm) treatment for 24 hours in serum-free RPMI media triggered the upregulation of the proinflammatory cytokine genes (TNF-α and IL-1β) in these cells (Fig 3B).

### Hyperosmolarity induces synchronous expression of AQP5 and Muc5ac in mouse conjunctival epithelial primary cells

**Fig 4:**
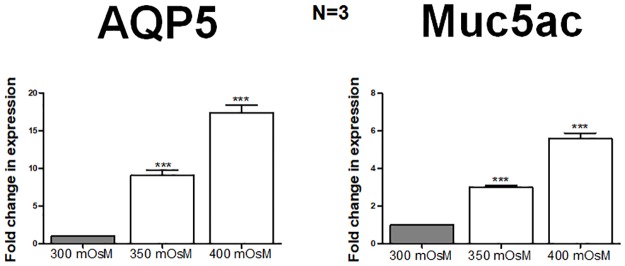
Hyperosmolarity induces AQP5 and Muc5ac expression in mouse conjunctival epithelial primary cells. Increase in the mRNA expression of AQP5 and Muc5ac in mouse conjunctival epithelial primary cells under hyperosmotic conditions (350 mOsm and 400 mOsm). A synchronous relationship in expression as triggered by hyperosmolarity was seen.

The mouse conjunctival epithelial primary cells incubated for 24 hours in hyperosmolar (350 and 400 mOsm) serum-free RPMI media showed a significantly elevated, synchronous expression of AQP5 and Muc5ac at mRNA level (Fig 4). The AQP5 mRNA showed a 9-fold upregulation (P = 0.0003) at 350 mOsm and 17-fold (P = 0.0001) at 400 mOsm. The Muc5ac mRNA was 3-fold up regulated (P = 0.0001) at 350 mOsM which increased to >5-folds (P = 0.0001) at 400 mOsM. This demonstrates that in mouse conjunctival epithelium, AQP5 and Muc5ac respond to external stimulus similarly to that in rabbits.

## Discussion

In our rabbit model, ocular inflammation associated with acute DE condition spontaneously resolved. We found that adequate tear is maintained by the conjunctiva through predominantly AQP5-mediated fluid secretion [13,23]. The present study focuses on the change of goblet cell-specific MUC5AC in the same model, and found its expression significantly elevated and comparable to that of AQP5. The expression of AQP5 and MUC5AC in rabbit conjunctival epithelium is in synchrony, with increased expression of both in response to the acute surgically-induced DE stress. Further, co-immunoprecipitation of AQP5 and MUC5AC suggests that they possibly interact with each other in one protein complex. The over expression of MUC5AC likely contributes to the compensatory restoration of ocular surface homeostasis. Distinct mouse ocular anatomy makes it nearly impossible to create a mouse DE model with complete surgical excision of the main LG and HG. We therefore used mouse conjunctiva primary epithelial cells and observed the same synchronous relationship between AQP5 and Muc5ac when grown under hyperosmotic stress (simulated DE condition). This indicates that such a coordinated response between AQP5 and MUC5AC is not specific to rabbits. To the best of our knowledge, this is the first time that such a synchronous relationship has been observed between the two molecules in the conjunctival epithelium. Our data further implies that a possible compensatory mechanism exists in the conjunctiva in acute DE condition.

It has been shown that DE related inflammatory mediators (TNFα, IL-1β, IL6/IL17, and prostaglandin E2) upregulate MUC5AC mRNA expression [33]. The simultaneous upregulation of AQP5 and MUC5AC has also been observed in lung adenocarcinoma cell line [17,18]. We believe such a relationship is biologically significant and should be distinguished from a chronically diseased condition. In airways of COPD patients, decreased AQP5 expression has been reported to be associated with increased MUC5AC expression [19]. Similarly in chronic DE condition, goblet cell number and MUC5AC content are reportedly reduced as well [5,9,10]. It is possible that in chronic condition such as DE and COPD, the compensatory mechanisms is compromised, resulting in aberrant relationship between AQP5 and MUC5AC expression. Further, Stephen et al suggested that inconsistent reports of mucin expression in DE disease could also be due to limited sample volume and variety of etiologies/disease severity [34]. In a desiccation-induced DE mouse model, the same group found unchanged number of Muc5ac RNA transcripts in the conjunctiva although Muc5ac concentration decreased as assessed by ELISA [35]. Such a decrease in Muc5ac could be due to the entrapment of goblet cells in reaction to desiccation stress. Moreover, DE in autoimmune conditions such as SS is marked by conjunctival inflammation (manifested by inflammatory cell infiltrates) [36] which causes the loss of conjunctival goblet cells [37] (hence the reduction in MUC5AC). In addition, certain membrane spanning mucin genes reportedly are upregulated under DE condition as well [38,39]. For example, MUC1 mRNA level increased in tears of SS patients [38]. Similarly, mRNA/protein of MUC1 and MUC16 increased in post-menopausal women with non-SS DE [39] and in atopic keratoconjunctivitis [40].

Mucin production is regulated by controlling the rate of synthesis, rate of mucin secretion, and number of goblet cells present in the conjunctiva [41]. The compensation seen in our rabbit model suggests an increased mucin synthesis (over-expression) as a mechanism. We are not able to assess if rate of mucin secretion has increased AE but we did not find significant change in goblet cell number BE and AE [13]. The co-immunoprecipitation study demonstrated that in the conjunctiva, AQP5 and MUC5AC possibly interact with each other. To the best of our knowledge, this has not been reported previously and could imply that interaction between these two epithelial proteins is important for MUC5AC to acquire its full function. An extended, stiff mucin polymer occupies a large hydrodynamic volume, and thus contributes to the rheological properties of a gel or solution and to the formation of a physiochemical barrier [42]. Mucosal surfaces are hydrated continuously by fluid secretions. Increased mucus solids and mucin concentrations increase the osmotic pressure and affect the distribution of water between the mucus and epithelial layer [43]. Defective fluid secretion and/or hyperabsorption would increase the concentration of the mucin molecules and the elevated osmotic pressure eventually causes adhesion and possibly immobilization of the mucus layer to the epithelial surface [44]. We thus hypothesize that presence of AQPs in proximity to and even interaction with MUC5AC on epithelial cell membrane are critical to ensure an adequately hydrated mucus gel, its full function and normal rheological properties. It is known that mucin proteins are densely packed and stored in secretory granules at low pH in an ionic background dominated by calcium in goblet cells [45]. Upon release, secretory granules undergo very rapid expansion in 3 to 6 seconds when calcium starts the process of exchanging to sodium [46]. Increased sodium concentration inside the gel raises the osmotic pressure, and water molecules move into the core of the highly organized gel, causing it to expand (swelling) [46]. Therefore, it is possible that the immediate availability of adequate water molecules is critical for mucin granules to unpack to the extended form, and the interaction between MUC5AC and AQP5 serves to this process.

Diquafosol (activator of P2Y2 receptor) stimulates mucin secretion from conjunctival goblet cells [47–49] and activates the fluid pump mechanism of the conjunctival accessory lacrimal glands [50–52], thus promoting stability of TF and rehydration [53]. Human clinical studies have demonstrated its efficacy in improving aqueous tear secretion [50,54,55] and mucin secretion [56–58]. Similarly, Rebamipide (quinolinone derivative) and JBP485 promoted increased MUC5AC expression and accelerated aqueous tear secretion [21,22].

Though the precise signaling pathway of ocular surface homeostasis restoration in the post-surgical rabbits is not yet defined, our data indicates that conjunctival AQP5 and MUC5AC mediated tear fluid and mucin compensation plays a central role. Thus regulatory mechanisms and the functional importance of this interaction need further exploration. The possible regulatory signaling pathway for upregulation of conjunctival AQP5 and MUC5AC could be through mitogen-activated protein kinase (MAPK) / extracellular signal-regulated kinase (ERK) signaling pathway. MAPK pathway is known to get activated by cellular stress [59]. Specifically, the c-Jun N-terminal kinase (JNK) and p38 MAPK classes of MAPK are known to be activated in response to inflammatory cytokines [60]. The DE condition causes ocular hyperosmolarity and inflammation [61,62] resulting in release of proinflammatory cytokines [13,23]. An increased pro-inflammatory cytokine levels can possibly activate c-Jun N-terminal kinase (JNK) or p38 MAPK pathway which further induces AQP5 upregulation. Expression of AQPs responds to hyperosmotic/hypertonicity condition known to be regulated by MAPK pathway [63–65]. In fact, hypertonic induction of AQP5 is reported to occur through an ERK-dependent pathway [63]. Further, cells highly expressing AQP5 can potentially cause the upregulation of MUC5AC through epidermal growth factor receptor (EGFR) signaling pathway. In human pulmonary adenocarcinoma cell lines, MUC5AC mucin expression is induced by AQP5 partly through EGFR signaling pathway [17].

The above hypothesized MAPK signaling pathway for upregulation of conjunctival AQP5 and MUC5AC can be experimentally tested by evaluating the expression of AQP5 and Muc5ac in mouse primary conjunctival epithelium cells grown under hyperosmotic conditions with different inhibitors of MAPK pathway such as SP600125 (JNK inhibitor), U0126 (ERK inhibitor) and SB203580 (p38 MAPK inhibitor). The reduced expression of AQP5 in these cells in the presence of MAPK inhibitors will imply the involvement of the MAPK pathway. Similarly, AG1478 (EGFR inhibitor) can be tested in the cells to assess the involvement of EGFR pathway in upregulation of Muc5ac.

To summarize, our data shows that conjunctival epithelium AQP5 and MUC5AC are expressed in synchrony in the mixed-mechanism DE condition surgically created in rabbits [13]. AQP5 has been regarded as an apical homologue [66] that could serve as a potential target for pharmacological upregulation [67]. Similarly, novel therapeutic approaches to treat ocular anomalies involving modulating goblet cell mucin production have also been previously discussed [68]. The purinergic agonists have been shown to stimulate both mucin secretion by conjunctival goblet cells [47] and transepithelial fluid movement [69], and have been demonstrated to ameliorate DE complications in individuals with LG dysfunction [51]. Our study further supports the notion that augmentation of the expression/function of AQP5 and MUC5AC can be an effective therapeutic modality for DE.
